# Ki-67 and overall survival in patients with glioblastoma: A systematic prognostic review and meta-analysis

**DOI:** 10.1093/noajnl/vdag111

**Published:** 2026-04-27

**Authors:** Ida Kaalhus Nordahl, Claes Johnstad, Ole Solheim, Sverre Helge Torp

**Affiliations:** Department of Clinical and Molecular Medicine, Faculty of Health and Medicine, Norwegian University of Science and Technology (NTNU), Trondheim, Norway; Department of Neuromedicine and Movement Science, Norwegian University of Science and Technology, Trondheim, Norway (C.J., O.S.); Department of Neuromedicine and Movement Science, Norwegian University of Science and Technology, Trondheim, Norway (C.J., O.S.); Department of Neurosurgery, St. Olav’s Hospital, Trondheim University Hospital, Trondheim, Norway; Department of Clinical and Molecular Medicine, Faculty of Health and Medicine, Norwegian University of Science and Technology (NTNU), Trondheim, Norway; Department of Pathology, St. Olav’s Hospital, Trondheim University Hospital, Trondheim, Norway

## Abstract

**Background:**

Ki-67 labeling index (LI) is widely used to quantify tumor proliferation in cancer. Although helpful in assessing proliferative activity across gliomas, its prognostic value in glioblastoma remains uncertain.

**Methods:**

We conducted a systematic prognostic review and meta-analysis of studies reporting associations between Ki-67 LI and overall survival in adult glioblastomas. Random-effects models (restricted maximum likelihood with Knapp-Hartung adjustments) were used to pool hazard ratios (HRs). Risk of bias was assessed with the QUIPS tool.

**Results:**

Fourteen retrospective cohort studies (*n* = 1,097) met the inclusion criteria. Across all studies, higher Ki-67 LI was associated with an increased hazard of death (univariable pooled HR 1.73, 95% confidence interval [CI] 1.33-2.25; *P* = .001; multivariable pooled HR 2.23, 95% CI 1.67-2.96; *P* = .0001). Prediction intervals were 0.99-3.02 (univariable) and 1.11-4.48 (multivariable), suggesting that future multivariable studies are more likely to observe HRs >1 in patients with higher Ki-67 LI. Between-study heterogeneity was low to moderate (*I*^2^ = 24.1% univariable; *I*^2^ = 40.3% multivariable). QUIPS assessments highlighted greatest concerns for selective study participation and heterogeneous Ki-67 cutoffs, while outcome measurement, confounder adjustment, and statistical reporting were generally lower risk.

**Conclusions:**

Higher Ki-67 LI is associated with an increased hazard of death in patients with glioblastoma, with effects persisting after multivariable adjustment and limited between-study heterogeneity. Standardized Ki-67 assessment and clinically validated cutoffs are needed to improve comparability and enable robust prognostic stratification.

Key Points

- Higher Ki-67 LI is associated with an increased hazard of death in patients with glioblastoma.
- Across studies, Ki-67’s prognostic estimates showed low heterogeneity, while Ki-67 LI cut-offs varied considerably.

Importance of the Study

Ki-67 labeling index (LI) is routinely used as a marker of proliferation in diffuse gliomas, yet its prognostic value in glioblastoma remains debated. By synthesizing available prognostic evidence, this systematic review and meta-analysis shows that higher Ki-67 LI is consistently associated an increased hazard of death in patients with glioblastoma. Effects persisted after multivariable adjustment and with low-to-moderate between-study heterogeneity. These findings support that Ki-67 provides prognostic information even within glioblastoma (WHO grade 4), beyond its role in broader glioma grading. However, substantial variation in Ki-67 cutoff values and concerns about selective study participation limit comparability and clinical translation. Standardized Ki-67 assessment and clinically validated cutoffs are needed to enable robust risk stratification and to support future prognostic and treatment-stratification studies.

Mitotic figures are commonly observed in glioblastoma tissue and have been used to distinguishing lower- from higher-grade tumors (WHO grades 1-4).^1^^,^^2^ Although mitotic figures are not a part of the integrated diagnosis of glioblastoma in the 2021 WHO classification, they remain a simple and relevant measure of tumor proliferation.^3^^,^^4^

Ki-67, commonly detected in formalin-fixed paraffin-embedded (FFPE) tissue using the MIB-1 antibody, is a widely used marker of proliferative activity in many human malignancies.^5^^,^^6^ It reduces observer bias in assessing mitotic figures, and the Ki-67 labeling index (LI) quantifies the proportion of actively dividing (non-G0) cells.^5^ While Ki-67 LI has shown value in stratifying prognosis across gliomas,^7^ the extent to which variation in Ki-67 LI within glioblastoma patients correlates with overall survival remains uncertain. This may be partly explained by the significant intertumoral and intratumoral heterogeneity of glioblastomas, which complicates interpretation of histological markers and contributes to variation in reported prognostic value across studies.^8^^,^^9^ When investigating the Ki-67 LI in glioblastoma patients, some question whether it truly is an independent predictor of survival.^10–19^ Some have found an increased Ki-67 LI to be associated with a better prognosis,^13^^,^^20^^,^^21^ while others claim that it is associated with a poorer prognosis.^18^^,^^22–27^

Given the variation in reported findings and the IDH-based definition of glioblastoma since 2021, a systematic review and meta-analysis may aid in clarifying the prognostic role of Ki-67 LI. In 2015 Chen et al and Thuy et al conducted two respective systematic reviews and meta-analyses on the prognostic value of Ki-67 in gliomas, and they found a weak but significant difference in survival between patients with high versus low expression of Ki-67.^7^^,^^28^ Considering evolving diagnostic criteria and the ongoing search for prognostic markers to guide treatment, the objective of this systematic review is to assess the current evidence for Ki-67 LI as a prognostic marker of overall survival in glioblastoma patients.

## Methods

### Eligibility Criteria

This systematic review was conducted and reported in accordance with the PRISMA 2020 (Preferred Reporting Items for Systematic Reviews and Meta-Analyses) guidelines.^29^

Studies with all the following criteria were included:

1. A study cohort including samples from adult (≥18 years), human, IDH-wildtype glioblastomas. To avoid exclusion of important literature, all studies with a clear dominance of IDH-wildtype glioblastomas in their data (≥75% of the patients) were included. This was to provide a cohort that would be relevant to the WHO 2021 classification of CNS tumors. An IDH-wildtype rate of at least 75% represents a substantial majority of the cohort and is comparable to the equivalent rate of the CBTRUS report (82.1%).^30^
2. Presenting LI of Ki-67 by immunohistochemistry or quantified expression of Ki-67.
3. Including analysis of Ki-67 in relation to overall survival, presented with a hazard ratio and confidence interval.

Exclusion criteria were:

1. Non-English written articles.
2. Full text unavailable after reasonable retrieval attempts.
3. No specification of IDH-analysis of the cohort.
4. Articles published prior to 2008 (when the importance of IDH was first published).^31^
5. Case reports.
  To enhance the quality and reliability of the meta-analysis, two post hoc exclusion criteria were applied:
6. Inclusion of recurrent tumors.
7. Ki-67 analyzed as a continuous variable without dichotomization.

These steps ensured comparability and valid effect estimation across studies.^32^

For synthesis of the data, studies were grouped based on whether the analyses were univariable or multivariable.

### Search Strategy and Information Sources

We selected “Ki-67 antigen,” “MIB-1 antibody,” and “Glioblastoma” as the main search concepts. The search was conducted in PubMed, Embase, and Web of Science. Both concepts had a corresponding thesaurus in PubMed and Embase. The respective thesaurus and free-text terms for each concept (found in Online Appendix S1), were combined using the Boolean operator OR. The final search, combining “Ki-67 antigen,” “MIB-1 antibody,” and “Glioblastoma” was executed using the Boolean operator AND. No other restrictions were used. The search for both Embase and PubMed was conducted on May 6, 2025 and updated on July 7, 2025. The search in Web of Science was conducted on June 6, 2025 and updated on July 7, 2025. The full search strategies for each database can be found in Supplementary Tables S1-S3.

### Selection Process

Two blinded evaluators (I.K.N. and C.J.) evaluated the articles based on their title and available abstract preview within each database. Articles that appeared potentially relevant based on title were then screened at the abstract level and exported to EndNote, where full texts were retrieved and read in full. No automation tools were used in the screening process. Discrepancies after full-text screening were resolved through discussion to reach consensus on final inclusion.

### Data Collection Process and Risk of Bias Assessment

We extracted univariable and multivariable hazard ratios (HRs) with 95% confidence intervals (CIs) relating Ki-67 LI to overall survival (OS), together with clinical variables (sample size, age, sex, performance status, extent of resection, radiotherapy, chemotherapy, and MGMT promoter methylation). Two reviewers (I.K.N. and C.J.) independently performed data extraction on half the studies and then cross-checked each other’s study sets. Disagreements were resolved by discussion. No automation tools were used.

Risk of bias was assessed with the QUIPS tool, following guidance from Brignardello-Petersen et al.^33^^,^^34^ To contextualize potential selection bias, we compared study demographics with reference distributions from CBTRUS (age, sex, and IDH-wildtype frequency),^35^ and we used Pham et al^36^ for treatment patterns and MGMT methylation rates. Outliers were defined as ±1 standard deviation (SD) from reference values for continuous variables and by pragmatic, consensus-based thresholds for categorical variables. When clarification was required, corresponding authors were contacted; unresolved items were adjudicated by consensus.

### Data Synthesis and Analysis

We pooled effect sizes using a random‐effects model with inverse‐variance weighting on log‐transformed hazard ratios (HRs). Between‐study variance (*τ*^2^) was estimated via restricted maximum likelihood,^37^^,^^38^ and 95% confidence intervals for pooled effects were calculated with Knapp-Hartung adjustments.^39^^,^^40^ Heterogeneity was summarized with *I*^2^ (from Cochran’s Q). We also reported a t-distribution–based prediction interval. Analyses were conducted in RStudio (version 2025.05.1 + 513) using the meta package.^41^

We extracted both univariable and multivariable HRs with 95% CIs wherever available. For one study (de Godoy et al^32^^,^^42^), HRs were derived by fitting Cox models from reported data. A priori, we planned subgroup analyses by IDH status: (1) IDH-wildtype glioblastoma; and (2) mixed cohorts (IDH-mutant and IDH-wildtype) where multivariable models adjusted for IDH. However, we chose to present a single pooled estimate because all included cohorts comprised ≥80% IDH-wildtype glioblastoma, and all studies with mixed IDH status (glioblastoma and astrocytoma grade 4) provided multivariable analyses that adjusted for IDH status. This strategy nearly doubled the number of studies included in the meta-analysis, improving precision; consequently, any residual confounding by IDH status is expected to be minimal. Nevertheless, two forest plots of only the studies that included 100% IDH-wildtype are presented in Supplementary Figures S1 and S2. Post hoc, to enhance comparability, we grouped studies by commonly used Ki-67 LI cut-offs, yielding threshold strata of 19%-20% (*n* = 5),^23^^,^^43–46^ and 15% (*n* = 4).^41^^,^^47–49^  *P* values <.05 were considered significant.

## Results

### Study Selection

**Figure 1.**
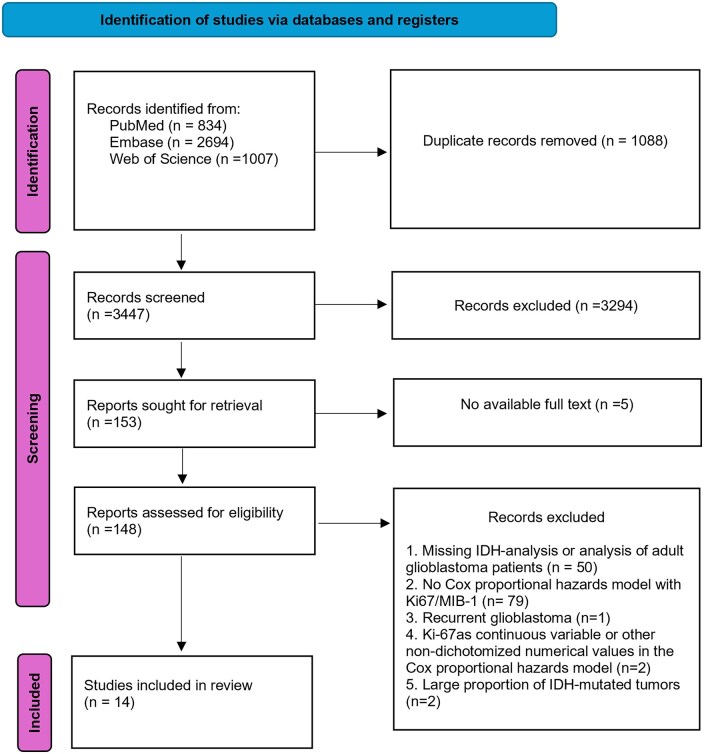
PRISMA flowchart for the study selection process.^29^ This work is licensed under CC BY 4.0.

A total of 3,447 unique records were identified for screening from the initial search in the three respective databases, PubMed, Embase, and Web of Science. Out of the 148 reports assessed for eligibility, 14 studies were included in this systematic review and meta-analysis (see PRISMA flowchart in Figure 1).

### Study Characteristics

#### Clinical data

A total of 14 retrospective cohort studies met the inclusion criteria.^10^^,^^23^^,^^41^^,^^43–54^ The studies were published between 2012 and 2025 and included data on 1097 patients with glioblastoma. Four studies reported data from China,^43^^,^^49^^,^^50^^,^^52^ four from Japan,^44^^,^^51^^,^^53^^,^^54^ two from Germany,^23^^,^^47^ and one each from India,^46^ Italy,^48^ South Korea,^45^ and the United States of America.^41^  Table 1 shows the categorization of the clinical characteristics reported in each study based on how they align with the reference values from the U.S. population-based cohorts from CBTRUS and Pham et al.^35^^,^^36^ The proportion of patients receiving both radiotherapy and temozolomide is reported in the table as ‘chemoradiotherapy’ (Stupp protocol or equivalent). The reference article by Pham et al^36^ does not specify whether this was according to the Stupp regimen; however, since most included studies explicitly reported treatment according to the Stupp protocol, these values were considered the closest approximation to chemoradiotherapy.

**Table 1.** Clinical data from the 14 included studies compared to reference values from two large U.S. population-based datasets

Clinical data (reference-value, SD)	Amount of the included studies	Sources
Median age (65, [57-72])		
>72	0	-
57-72	2	^41^ ^,^ ^52^
< 57	2	^43^ ^,^ ^46^
Mean age (no reference dataset)		
>72	0	-
57-72	5	^23^ ^,^ ^44^ ^,^ ^47^ ^,^ ^51^ ^,^ ^53^
< 57	3	^45^ ^,^ ^49^ ^,^ ^50^
Missing data, age	2	^48^ ^,^ ^54^
Percentage of males (59.3%, ±5%)		
>64.3	4	^23^ ^,^ ^41^ ^,^ ^46^ ^,^ ^49^
54.3-64.3	8	^43–45^ ^,^ ^47^ ^,^ ^51–54^
< 54.3	1	^50^
Missing data, males	1	^48^
Percentage of IDH-wildtype (80.4%, ±5%)		
100	7	^23^ ^,^ ^41^ ^,^ ^47^ ^,^ ^48^ ^,^ ^50^ ^,^ ^53^ ^,^ ^54^
>85	6	^44–46^ ^,^ ^49^ ^,^ ^51^ ^,^ ^52^
75-85	1	^43^
<75	0	-
Median overall survival (9 months, [8-9])*		
>9	9	^41^ ^,^ ^45–50^ ^,^ ^52^ ^,^ ^53^
8-9	0	-
< 8	0	-
Median overall survival (12.4 months [12.2, 12.6])**		
>12.6	6	^41^ ^,^ ^45–47^ ^,^ ^49^ ^,^ ^53^
12.2-12-6	2	^48^ ^,^ ^50^
< 12.2	1	^52^
Missing data, survival	5	^23^ ^,^ ^43^ ^,^ ^44^ ^,^ ^51^ ^,^ ^54^
Percentage of gross total resection (GTR) (41.2%, ±5%)		
>46.2	9	^41^ ^,^ ^43^ ^,^ ^45–47^ ^,^ ^49–52^
36.2-46.2	0	-
<36.2	2	^44^ ^,^ ^48^
Missing data	3	^23^ ^,^ ^53^ ^,^ ^54^
Percentage of patients receiving chemoradiotherapy (Stupp or equivalent***) (72.7%), ±5%)		
>77.7	3	^44^ ^,^ ^47^ ^,^ ^48^
67.7-77.7	1	^23^
< 67.7	3	^46^ ^,^ ^50^ ^,^ ^52^
Missing data	7	^41^ ^,^ ^43^ ^,^ ^45^ ^,^ ^49^ ^,^ ^51^ ^,^ ^53^ ^,^ ^54^
Percentage of patients with high KPS		
≥50	9	^23^ ^,^ ^43^ ^,^ ^45^ ^,^ ^47–49^ ^,^ ^51–53^
<50	1	^46^
Missing data	4	^41^ ^,^ ^44^ ^,^ ^50^ ^,^ ^54^
Percentage of MGMT-methylated tumors (30.9%, ±5%)		
>35.9	6	^23^ ^,^ ^44^ ^,^ ^47^ ^,^ ^48^ ^,^ ^52^ ^,^ ^54^
³25.9-35.9	3	^41^ ^,^ ^49^ ^,^ ^50^
< 25.9	0	-
Missing data	5	^43^ ^,^ ^45^ ^,^ ^46^ ^,^ ^51^ ^,^ ^53^

Age, sex, and IDH-wildtype frequencies were retrieved from the CBTRUS Statistical Report,^35^ while treatment and MGMT-status data were obtained from Pham et al.^36^ Thresholds for outliers were based on ±1 SD from the reports where applicable or pragmatically defined. We included reference values for median overall survival from both the CBTRUS Statistical Report and the U.S. National Cancer Database as reported by Pham et al.^35^^,^^36^

Abbreviations: SD: standard deviation; IDH: isocitrate dehydrogenase; KPS: Karnofsky performance status; MGMT: O6-methylguanine DNA-methyltransferase.

* Value reported from the CBTRUS-report by Price et al.^35^

** Value reported from for IDH-wildtype glioblastoma Pham et al.^36^

*** Data represent the proportion of patients receiving combined radiotherapy and temozolomide. In most included studies, this was specified as the Stupp protocol; in the reference cohort (72.7%, ±5%), the protocol was not specified. Minor deviations from the Stupp regimen may have occurred.

#### Ki-67 expression thresholds

Most studies used varying Ki-67 LI cutoff values to evaluate its prognostic significance in their Cox proportional hazards models, with thresholds ranging from 12.7%^51^ to 30%.^50^ A cutoff of 19%-20% was the most frequently used value (*n* = 5),^23^^,^^43–46^ followed by 15% (*n* = 4).^41^^,^^47–49^ All the studies that reported which antigen they had used (*n* = 7), sourced it from Agilent (formerly Dako).^10^^,^^23^^,^^43–45^^,^^48^^,^^51^^,^^54^ The same studies also stated how the Ki-67 LI was counted (see Supplementary Table S4).

### Synthesis of Results

#### Pooled effect size

**Figure 2.**
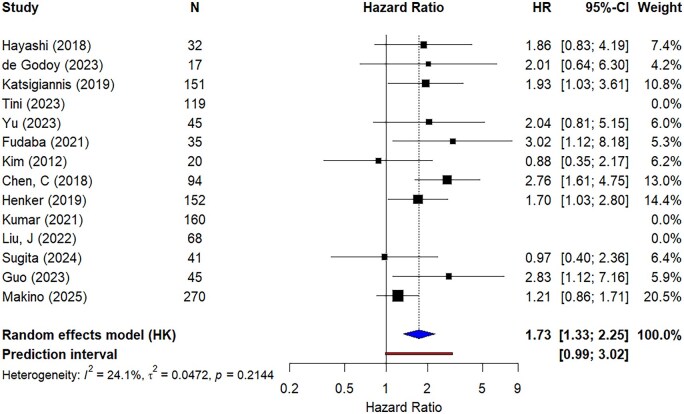
Forest plot showing the results of the meta-analysis of the univariable hazard ratios. HR: hazard ratio; CI: confidence interval.

**Figure 3.**
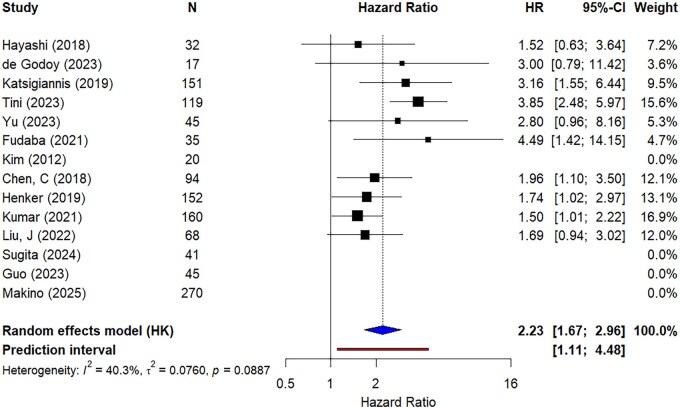
Forest plot showing the results of the meta-analysis of the multivariable hazard ratios. HR: hazard ratio; CI: confidence interval.

Figures 2 and 3 show the results of the meta-analyses of the pooled univariable and multivariable HRs, respectively. Both analyses show a higher hazard of death for patients with higher Ki-67 LI, with a pooled HR of 1.73 (95% CI: 1.33-2.25) for the univariable and 2.23 (95% CI: 1.67-2.96) for the multivariable analysis. A bubble plot that shows the effect estimates of the meta-analysis by Ki-67 LI cutoff can be found in Supplementary Figure S8. There was no apparent association between KI-67 cut offs and reported HRs.

#### Between-study heterogeneity

For the univariable analysis, the between-study heterogeneity variance was estimated at *τ*^2^ = 0.047 (95% CI: 0.000-0.361), with an *I*^2^ value of 24.1% (*P* value = .28, 95% CI: 0.0%-62.2%). The HR prediction interval (PI) ranged from 0.986 to 3.025. For the multivariable analysis, *τ*^2^ = 0.076 (95% CI: 0.000-0.386), *I*^2^ = 40.3% (*P* value = 0.056, 95% CI: 0.0%-71.5%), and the HR PI ranged from 1.106 to 4.483. Due to relatively modest heterogeneity in the results (*I*^2^ < 50%), we have not presented the subgroup analysis, but a GOSH (graphical display of study heterogeneity) plot and cluster analysis can be found in Supplementary Figures S4-S7).

#### Risk of bias in included studies

**Figure 4.**
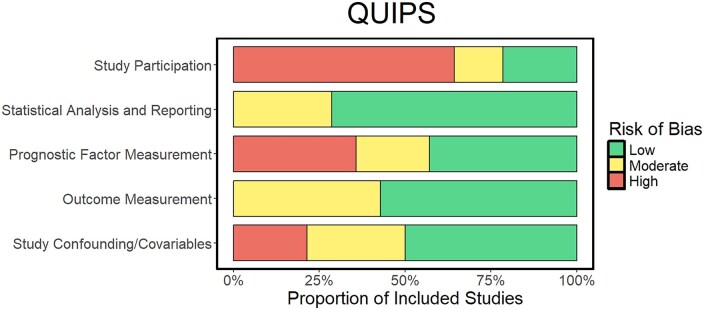
Bar plot showing the distribution of bias among the respective domains of the QUIPS tool.

The results of the QUIPS bias form are summarized in Figure 4, which shows the proportion of studies with low, moderate, or high risk of bias within the respective domains. The complete bias rating for each study can be found in Supplementary Figure S3. Evidently, most of the potential bias comes from patient selection, followed by measurement of Ki-67 LI and potential confounders. Outcome measurement and statistical analyses had lower risk of bias.

#### Findings in excluded studies

Some studies investigated the prognostic value of Ki-67 LI but were excluded from the meta-analysis due to the lack of reported hazard ratios or analysis of Ki-67 as a continuous variable. In the studies that lacked hazard ratios with confidence intervals, six studies found a significant inverse correlation between Ki-67 LI and overall survival,^22^^,^^55–59^ whereas one did not find any significant association.^60^ In the two studies that were excluded because they analyzed Ki-67 LI as a continuous variable, there was no significant association between Ki-67 LI and overall survival.^61^^,^^62^

## Discussion

This systematic review and meta-analysis provide evidence that the Ki-67 LI is an independent prognostic biomarker for overall survival in glioblastoma. Although there is no agreed cut-off or apparent gradient of effects across Ki-67 cut-offs, the observed pooled independent hazard ratio across studies was 2.23 (95% CI 1.67-2.96), indicating a prognostic impact higher than or at least comparable to established factors such as MGMT methylation status or gross total surgical resection.^63^ Thus, future prognostic models in glioblastoma should explore and presumably incorporate the Ki-67 LI.

Established prognostic factors in glioblastoma are heterogenous and mainly reflect patient-related factors such as age, functional status, or treatment related factors such as extent of surgical resection or chemo-/radiotherapy. With the exception of MGMT status which is a prognostic, but mainly a predictive factor,^64^ there is a paucity of treatment independent prognostic factors that reflect tumor biology. Ki-67 is readily available and may seem to harbor clinically meaningful prognostic value, independent of patient and treatment factors.

The between-study heterogeneity was low, and low-to-moderate for the univariable and multivariable pooled analyses, respectively, and were thus considered unlikely to influence the effect estimates. The PIs reflected the expected range of true effects (HRs) in comparable future studies. Notably, the univariable PI included values marginally below unity (HR = 1), implying that some future studies may observe no difference between patients with high versus low Ki-67 LI when no covariables are adjusted for. By contrast, the multivariable PI did not include unity, making it more likely that future adjusted studies will report increased mortality risk with higher Ki-67 LI. Taken together with the low τ^2^ estimates, this indicated a low heterogeneity and a high probability that the observed association between higher Ki-67 LI and poorer outcome was consistent.

Even though there was no significant between-study heterogeneity, we did explore the moderate variation in the multivariable analysis. A GOSH plot showed that all combinations of the included studies would show an association between higher Ki-67 LI and shorter overall survival. Additional GOSH diagnostics pointed out two studies as potential outliers.^46^^,^^48^ Removing these studies would decrease the *I*^2^, but the effect estimate would remain approximately the same. Given the relatively low statistical heterogeneity, most studies point in the same direction and support the clinical relevance of the Ki-67 index for assessing overall survival in glioblastoma. The higher heterogeneity observed in the collective multivariable analysis is more plausibly attributable to differences in study design and potential biases, rather than true effect modification. The studies in the meta-analysis all used different covariables in their multivariable cox models, which could explain the higher heterogeneity in the multivariable, compared to that of the univariable, effect estimates. A table of the covariables included in the respective studies can be found in Supplementary Table S5.

Among the bias domains, study participation carried the highest risk of bias. Many studies enrolled patients with high KPS were more often eligible for aggressive treatment and showed longer overall survival than population benchmarks. The included studies applied predefined inclusion and exclusion criteria, yielding well-defined populations and more precise effect estimates. However, excluding patients with significant comorbidity or other complicating factors introduces a selection bias and reduces external validity.^65^ The second most frequent high-risk bias domain was measurement of the prognostic factor, Ki-67. As summarized in Supplementary Table S3, quantification procedures varied and several studies did not clearly report how Ki-67 LI was measured.^41^^,^^46^^,^^47^^,^^49^^,^^50^^,^^52^^,^^53^ When reported, all studies used the same primary antibody (Agilent), which reduces inter-antibody variability,^66^ but differences in scoring approach and counting methods likely remained a source of measurement bias. Arguably the most consequential design issue was the lack of consensus on a cutoff for dichotomizing Ki-67 LI. Many studies used cohort-specific medians or means or data-driven “optimal” thresholds. The use of study-specific cutoffs rather than a standardized threshold complicates cross-study comparability and prognostic stratification. Across the remaining domains, the risk of bias was minimal. However, two of the studies that did not find a significant HR for Ki-67 LI in univariable Cox models omitted the variable from the multivariable analyses, which again could explain why the effect estimate was stronger in the meta-analysis of the multivariable HRs.

The results of the current systematic review and meta-analysis suggest that Ki-67 LI is associated with overall survival in patients with glioblastoma. In the two previous systematic reviews and meta-analyses on the topic, both from 2015, they found a pooled HR of 1.67 and 1.21 in the high Ki-67 group, respectively,^7^^,^^28^ as compared to our univariable HR of 1.73 and multivariable HR of 2.23. Although the clinically relevant prognostic effect of Ki-67 is difficult to interpret from these numbers, the absolute differences in reported median overall survival between the low and high Ki-67 expression groups ranged from 1 to 19 months, as seen in Supplementary Table S6. Furthermore, with a baseline 1-year relative survival of 43% in glioblastomas,^30^ the theoretical 1-year relative survival in the high Ki-67 LI group-based on the observed multivariable HR of 2.23—would be 15% (95% CI: 8%-24%). The equivalent estimate for the univariable HR would be 23% (95% CI: 15%-33%). Evidently, the Ki-67 may be associated with considerable differences in the prognosis of glioblastoma patients. However, any further estimation of the clinical impact of Ki-67 Li would be difficult with the available data. Although the mechanism for this survival benefit is unclear, there are some hypotheses. Chen et al found that patients with a higher expression of Ki-67 had better effect of chemotherapy.^67^ This phenomenon is vastly studied in carcinomas of the breast, amongst others, where patients often are stratified for treatment according to their Ki-67 LI. In glioblastomas, however, the association between Ki-67 LI and treatment could be useful to study whether the same stratification could have prognostic implications for the patients.

As this meta-analysis indicates, Ki-67 LI is a prognostic factor for overall survival, but current methods for analyzing and reporting it are heterogeneous. A standardized cut-off value and uniform method of measuring Ki-67 could contribute to stronger evidence on its prognostic value and may in the future aid clinicians or researchers in patient stratification. Although a common cut-off value would standardize definitions, tissue sampling may also matter, as KI-67 can be underrepresented in small tissue samples.^8^ Despite the variations in cut-off values across the current literature, the current meta-analysis excluded studies that analyzed Ki-67 LI as a continuous variable. While such an eligibility criterion could factor out relevant research, there is a clear majority of studies that dichotomized the Ki-67 LI in the current literature, and combining these for a common meta-analysis would prove challenging in terms of comparability. Only two, otherwise eligible, studies were identified with a continuous analysis of Ki-67, and these studies found no significant association between Ki-67 and overall survival.^61^^,^^62^ Dichotomization of continuous variables may simplify the data and remove important nuances. However, identifying a clinically relevant cut-off value could provide improved interpretability of the analyses and be directly applied to prognostic stratification of glioblastoma patients.

The most notable biases of patient selection and Ki-67 measurement and dichotomization in the included studies were some of the most important limitations of this systematic review and meta-analysis. A related data-retrieval issue was that Ki-67 was often investigated as a secondary variable rather than a primary target of survival analysis, thus increasing the risk of missing or incompletely recorded data. This includes incomplete reporting of antibody clone and quantification methods, which may have affected the estimated strength of association. Additionally, hazard ratios were often not reported or not directly available,^67^^,^^68^ which constrained study inclusion and may have biased the pooled evidence toward better-reported analyses. Clinical heterogeneity between cohorts, including differences in adjuvant treatment, may also have influenced effect estimates. Although the study cohorts ideally should include only IDH-wildtype tumors, there were some older data that included IDH-mutant glioblastomas. However, the IDH-wildtype rate in the total cohort was 96%, and IDH mutation status was adjusted for in all multivariable models; additionally, meta-analyses of the studies that only included IDH-wildtype gliomas showed comparable results to those of the full-data analyses. The IDH-wildtype rates and methods for assessment of IDH status in the respective studies are reported in Supplementary Tables S7 and S8, respectively. Furthermore, relying on histopathological diagnosis alone could lead to misclassification of well-defined glioma subtypes. This is an important limitation, particularly to older studies from when investigation of molecular characteristics was less common. Hence, we have provided a Supplementary Table S9 with the reported diagnostic criteria across the respective studies included. The greatest strengths of the current study were the systematic study selection process by two independent authors, as well as the low heterogeneity of the included studies.

## Conclusion

In this systematic review and meta-analysis, higher Ki-67 LI was associated with shorter overall survival in patients with glioblastoma. These effects were consistent after multivariable adjustment and had a low-to-moderate between-study heterogeneity. Risk-of-bias assessment highlighted concerns related to study participation and Ki-67 measurement, especially concerning heterogeneous cutoffs. These findings support Ki-67 LI as a prognostic marker while underscoring the need for standardized assessment protocols and clinically validated cutoffs to improve comparability and enable robust risk stratification in future studies.

## Supplementary Material

vdag111_Supplementary_Data

## Data Availability

Data are available upon reasonable request.

## Protocol and Registration

The protocol for this study was published on 4 July 2025 in the PROSPERO database. It can be found at https://www.crd.york.ac.uk/PROSPERO/view/CRD420251065678.
