# Supplementary material for: Ki-67 and overall survival in patients with glioblastoma: A systematic prognostic review and meta-analysis
Source: Neurooncol Adv. 2026 Apr 27;8(1):vdag111. doi: 10.1093/noajnl/vdag111 (PMC13213614; doi:10.1093/noajnl/vdag111)
Supplement: vdag111_Supplementary_Data [file vdag111_supplementary_data.zip › Revised Supplementary materials.docx]

**SUPPLEMENTARY MATERIAL**

**Search strategy**

*Table 1, PubMed search*

| Row | Search | Results |
| --- | --- | --- |
| 1 | "Glioblastoma"[Mesh] | 36,728 |
| 2 | Glioblastoma* [tw] | 63,346 |
| 3 | GBMs[tw] | 2,560 |
| 4 | GBM[tw] | 26,274 |
| 5 | "Ki-67 Antigen"[Mesh] | 19,124 |
| 6 | "MIB-1 antibody" [Supplementary Concept] | 145 |
| 7 | "Ki-67*"[tw] | 37,521 |
| 8 | "MIB-1*"[tw] | 3,968 |
| 9 | “Ki 67”[tw] | 37,493 |
| 10 | “MIB 1”[tw] | 3,961 |
| 11 | Ki67*[tw] | 45,571 |
| 12 | MIB1*[tw] | 4,679 |
| 13 | "Glioblastoma"[MeSH] OR "glioblastoma*"[tw] OR "GBMs"[tw] OR "GBM"[tw] | 69,272 |
| 14 | "Ki-67 Antigen"[MeSH] OR "MIB-1 antibody"[Supplementary Concept] OR "ki 67*"[tw] OR "mib 1*"[tw] OR "Ki 67"[tw] OR "MIB 1"[tw] OR "ki67*"[tw] OR "mib1*"[tw] | 52,681 |
| 15 | (((("Glioblastoma"[Mesh]) OR (Glioblastoma*[tw])) OR (GBMs[tw])) OR (GBM[tw])) AND (((((((("Ki-67 Antigen"[Mesh]) OR ("MIB-1 antibody" [Supplementary Concept])) OR ("Ki-67*"[tw])) OR ("MIB-1*"[tw])) OR ("Ki 67"[tw])) OR ("MIB 1"[tw])) OR (Ki67*[tw])) OR (MIB1*[tw])) | 1,188 |
| 16 | (((("Glioblastoma"[Mesh]) OR (Glioblastoma*[tw])) OR (GBMs[tw])) OR (GBM[tw])) AND (((((((("Ki-67 Antigen"[Mesh]) OR ("MIB-1 antibody" [Supplementary Concept])) OR ("Ki-67*"[tw])) OR ("MIB-1*"[tw])) OR ("Ki 67"[tw])) OR ("MIB 1"[tw])) OR (Ki67*[tw])) OR (MIB1*[tw])) Filters applied: from 2008/1/1 - 2025/7/7 | 834 |

[Mesh] = Exploded Medical Subject Heading, [Supplementary Concept]= supplementary concept, [tw]= title, abstract and keywords.

*Table 2, Embase search*

| \| Row \| Search \| \| Results \| \| --- \| --- \| --- \| --- \| \| 1 \| \| 'glioblastoma'/exp \| 107,331 \| \| 2 \| \| glioblastoma*s \| 91,082 \| \| 3 \| \| gbms*:ti,ab,kw \| 5,586 \| \| 4 \| \| gbm*:ti,ab,kw \| 47,517 \| \| 3 \| \| 'ki 67 antigen'/exp \| 78,122 \| \| 4 \| \| 'mib 1 antibody'/exp \| 23 \| \| 5 \| \| 'ki-67*':ti,ab,kw \| 45,716 \| \| 6 \| \| 'mib-1*':ti,ab,kw \| 5,557 \| \| 7 \| \| 'ki 67':ti,ab,kw \| 45,647 \| \| 8 \| \| 'mib 1':ti,ab,kw \| 5,521 \| \| 9 \| \| ki67*:ti,ab,kw \| 79,215 \| \| 10 \| \| mib1*:ti,ab,kw \| 6,921 \| \| 11 \| \| #1 OR #2 OR #3 OR #4 \| 132,974 \| \| 12 \| \| #5 OR #6 OR #7 OR #8 OR #9 OR #10 OR #11 OR #12 \| 118,339 \| \| 13 \| \| #13 AND #14 \| 3,076 \| \| 14 \| \| #13 AND #14 AND [2008-2025]/py \| 2.694 \| \|  \|  \|  \|  \| |  |  |  |
| --- | --- | --- | --- | --- | --- | --- | --- | --- | --- | --- | --- | --- | --- | --- | --- | --- | --- | --- | --- | --- | --- | --- | --- | --- | --- | --- | --- | --- | --- | --- | --- | --- | --- | --- | --- | --- | --- | --- | --- | --- | --- | --- | --- | --- | --- | --- | --- | --- | --- | --- | --- | --- | --- | --- | --- | --- | --- | --- | --- | --- | --- | --- | --- | --- | --- | --- | --- | --- | --- | --- | --- | --- | --- | --- | --- |
|  |  |  |  |

/exp = Emthree term exploded, :ti,ab,kw = title, abstract and keywords

*Table 3, Web of Science search*

| Row | Search | Results |
| --- | --- | --- |
| 1 | ("glioblastoma" OR "glioblastomas" OR "GBM" OR "GBMs") AND ("Ki-67" OR "Ki 67" OR "Ki67" OR "MIB-1" OR "MIB 1" OR "MIB1" OR "MIB-1 antibody")  Publication Date: 2008-01-01 to 2025-07-07 | 1,007 |

**Forest plots for IDH-wildtype glioblastomas only**

**Univariable**


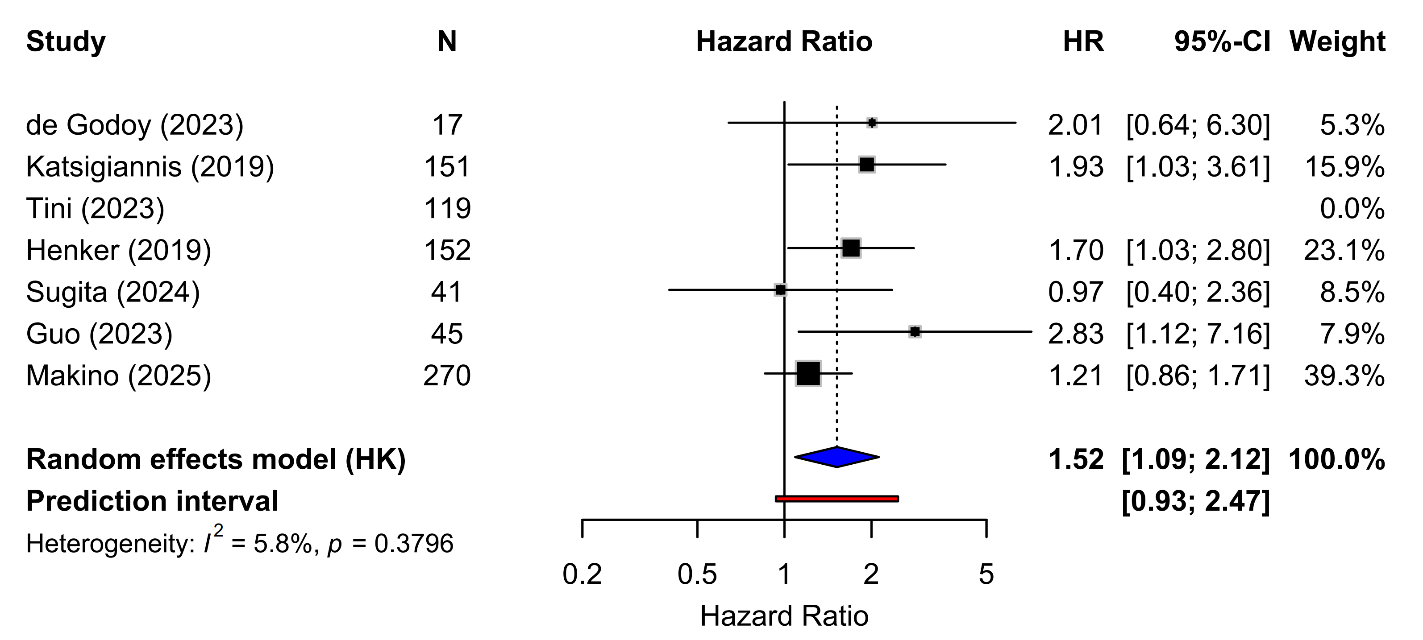


*Figure 1: Forest plot showing the results of the meta-analysis of the univariable HRs from studies that included IDH-wildtype glioblastomas only. HR: hazard ratio; CI: confidence interval.*

**Multivariable**


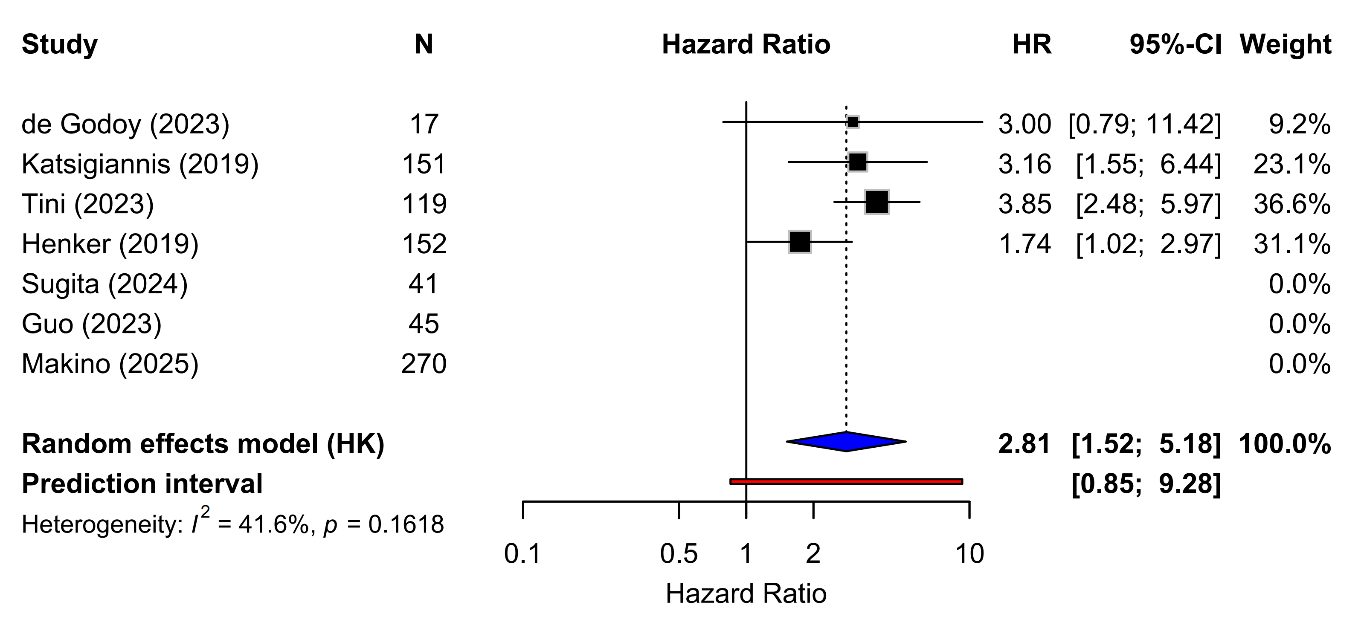


*Figure 2: Forest plot showing the results of the meta-analysis of the multivariable HRs from studies that included IDH-wildtype glioblastomas only. HR: hazard ratio; CI: confidence interval.*

**QUIPS form**


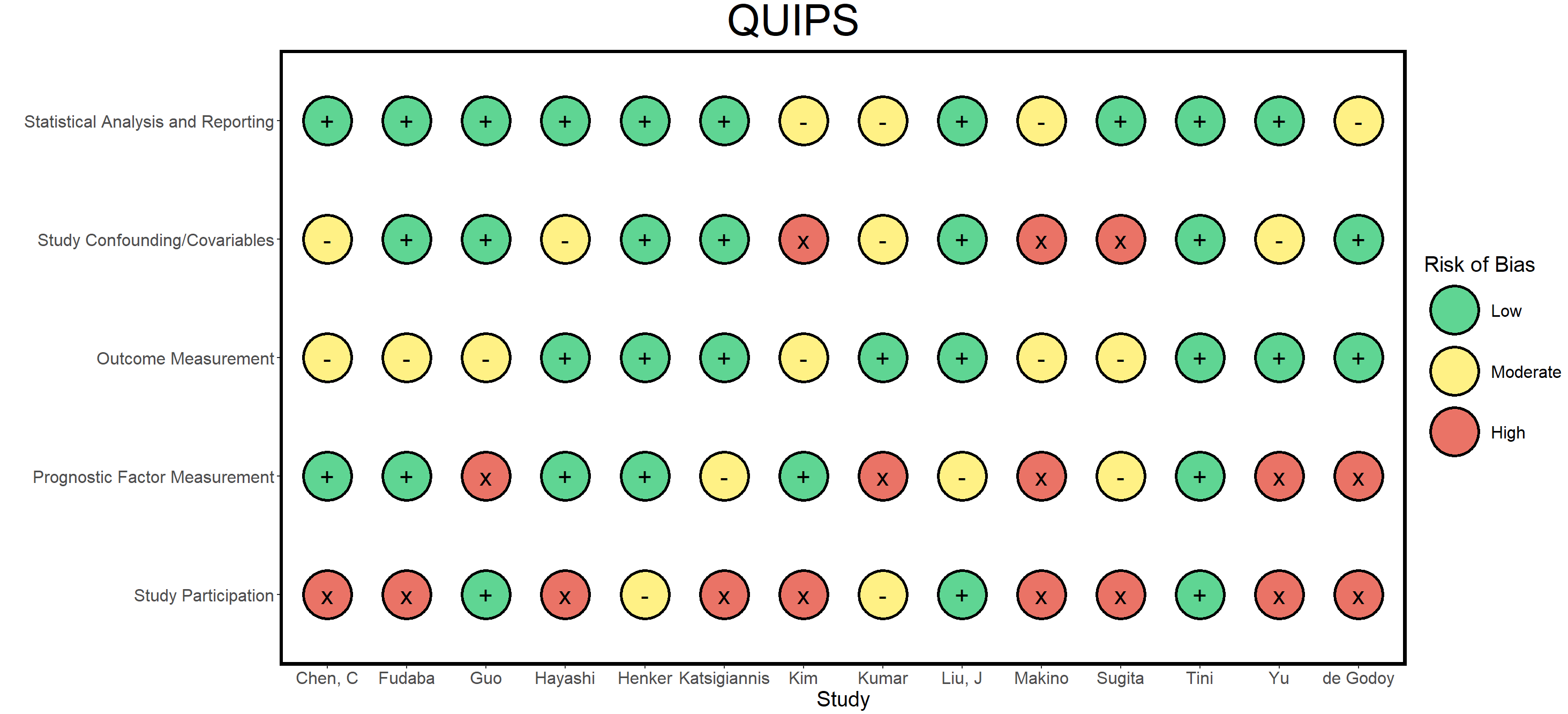


*Figure 3: Total QUIPS assessment for all the included studies with the risk of bias.*

**GOSH-plot**


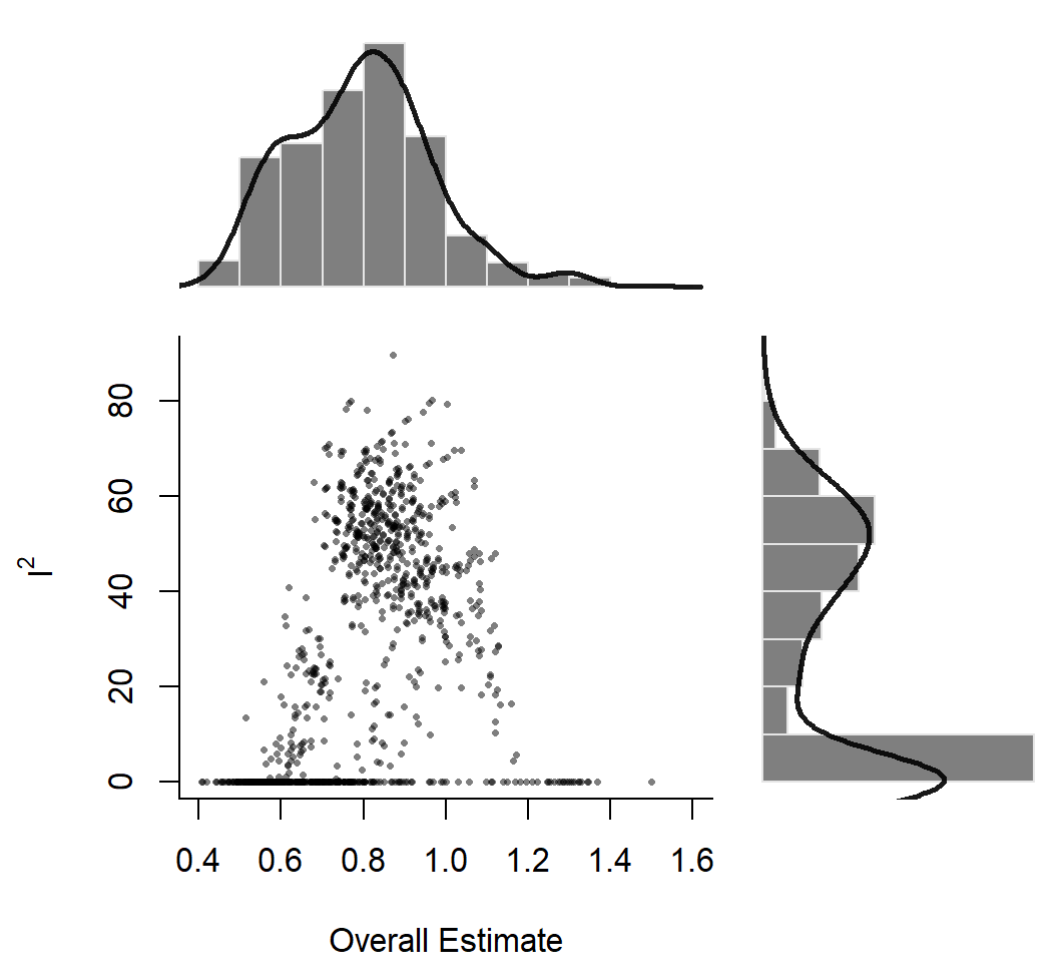


*Figure 4: GOSH-plot analysis showing graphical display of study heterogeneity showing I^2^ and log(HR) for different combinations of the included studies in the meta-analysis.*

**Outlier analysis**


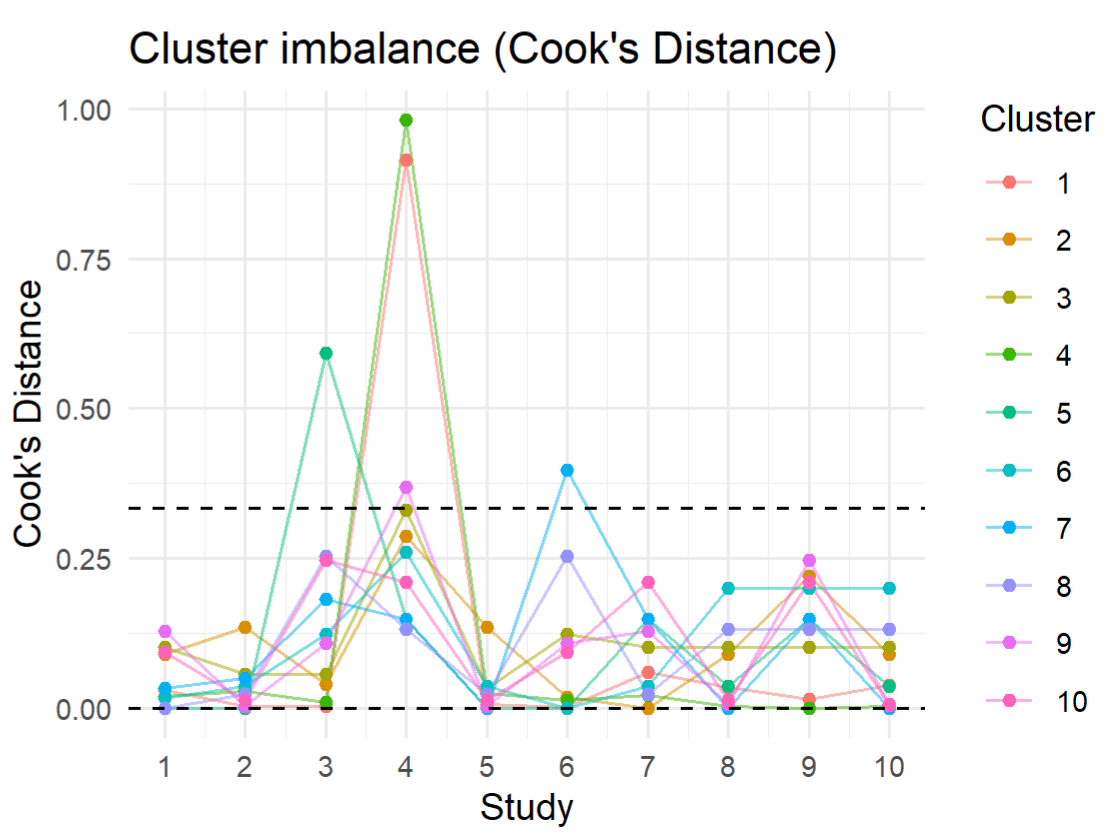


*Figure 5: DBSCAN Algorithm*


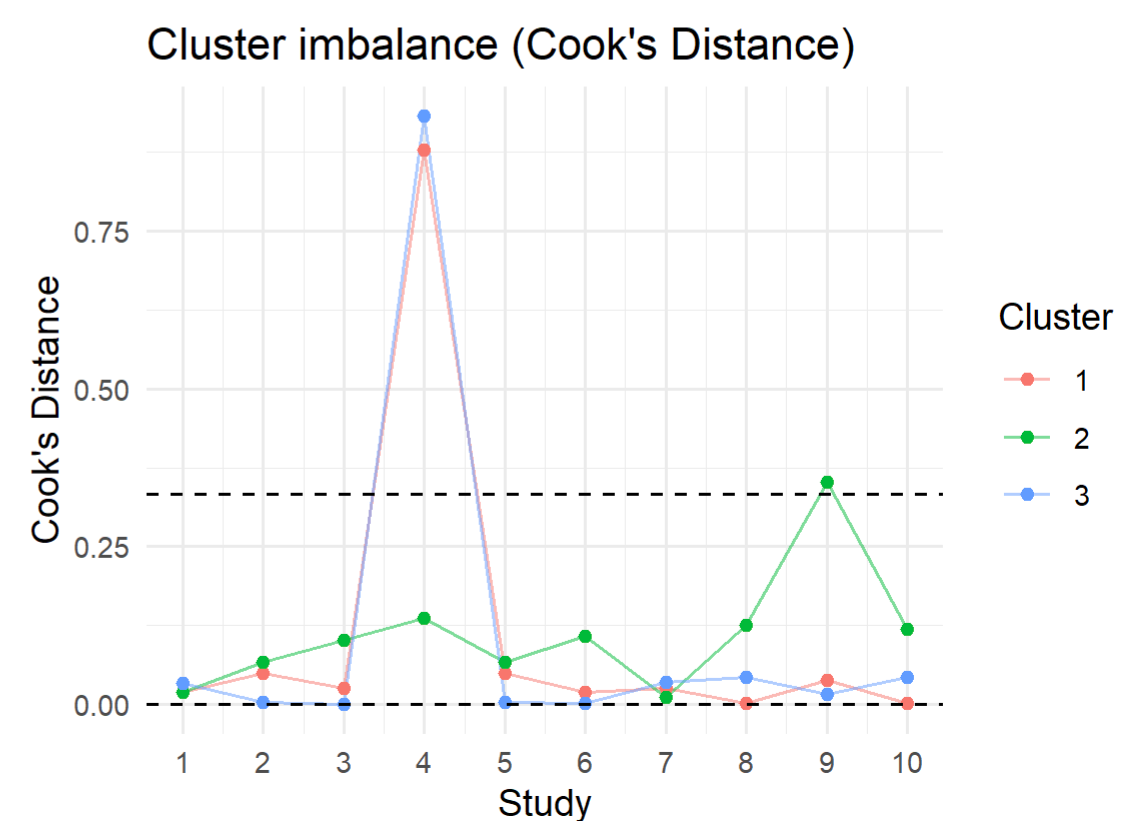


*Figure 6: K-means Algorithm*


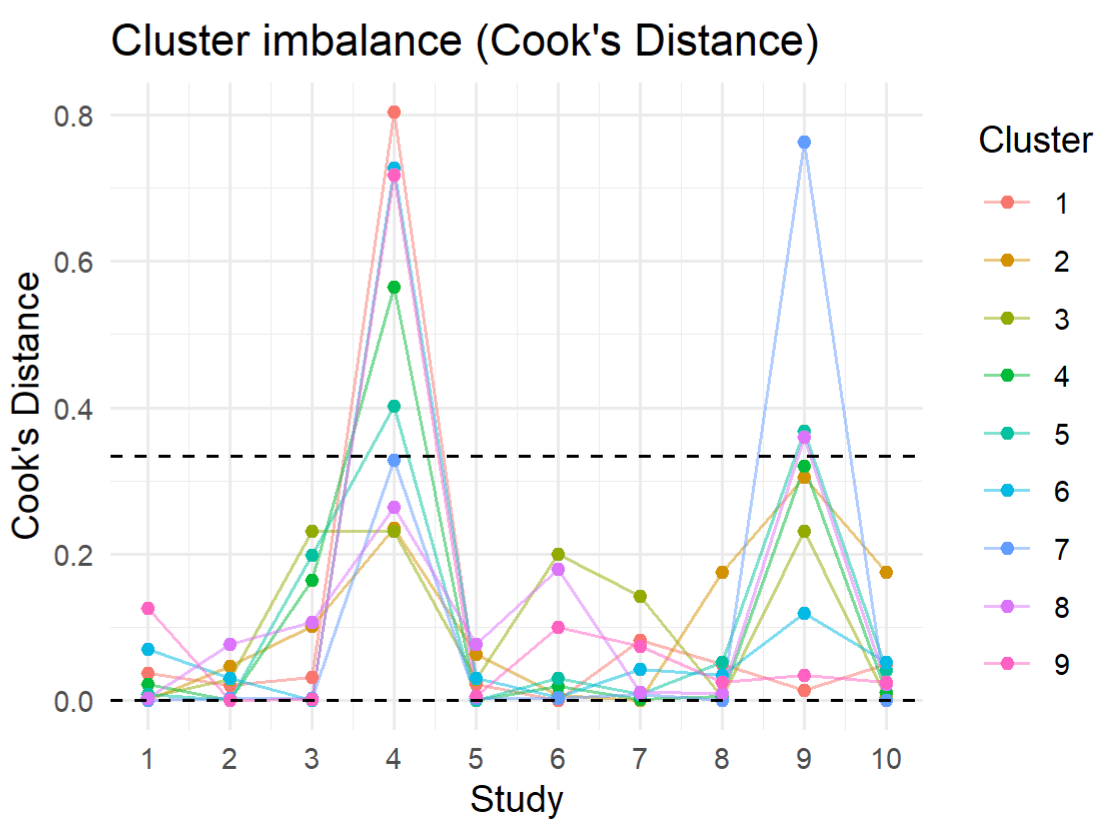


*Figure 7: Gaussian Mixture Model (GMM)*

Dotted line plot showing cluster imbalance, defined by Cook's distance, of the respective studies included in the meta-analysis. Three plots showing three different methods for uncovering outlier studies in a meta-analysis. There is one study that is pointed out in all three methods^1^ and one study that stands out in two of them.^2^

**Ki67- quantification methods**

Table 4, The reported method for Ki-67 quantification.

| **Author** | **Ki-67 quantification** |
| --- | --- |
| Chen, C et al. [41] | Percentage of Ki-67-positive tumor cells (positive nuclei/total tumor cells) in five randomly selected microscopic fields at 400× magnification with representative tumor tissue. |
| Fudaba et al. [42] | Percentage of Ki-6-positive tumor cells positive nuclei/total tumor cells) in ten randomly selected microscopic fields at 400× magnification. |
| Hayashi et al. [49] | Percentage of Ki-67-positive tumor cells (positive nuclei/total tumor cells) in five randomly selected microscopic fields at 200× magnification. |
| Henker et al. [23] | Percentage of Ki-67-positive tumor cells (positive nuclei/total tumor cells) in areas with the highest density of immunoreactivity, with a minimum of 1000 tumor cells or 3 hot spots at 200× magnification. Exclusion of necrotic areas and vascular endothelium. |
| Kim et al. [43] | Percentage of Ki-67-positive tumor cells (positive nuclei/total tumor cells) in five fields (hot spots) at 400× magnification, quantified semiautomatically using a computer image analysis system. |
| Sugita et al. [52] | Percentage of Ki-67-positive tumor cells (positive nuclei/total tumor cells) within a high power (40× objective) field. |
| Tini et al. [46] | Percentage of Ki-67-positive tumor cells (positive nuclei/total tumor cells) from all tumor cells. |

**Bubble plot**


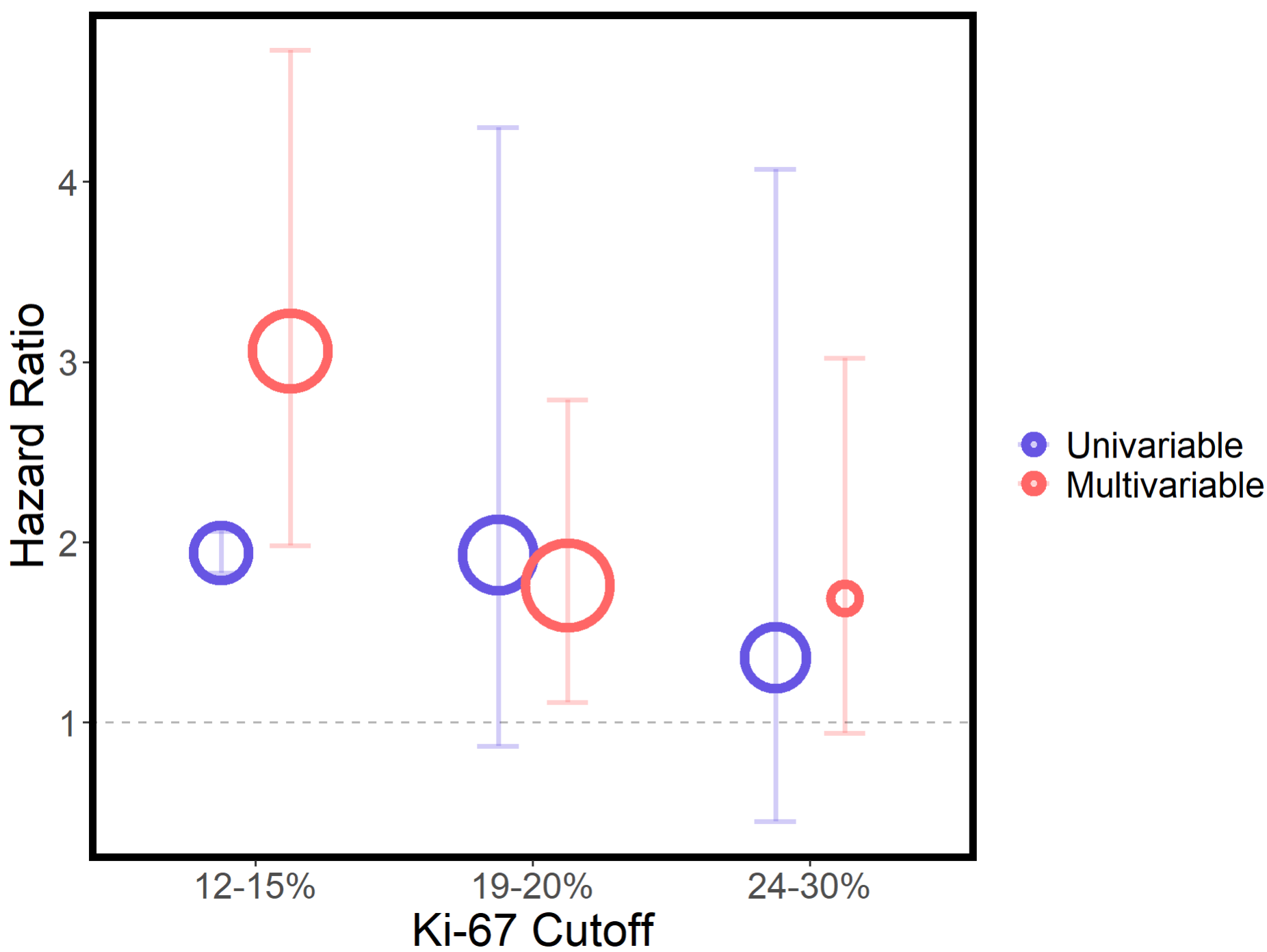


*Figure 8: Bubble plot showing the pooled effect estimates based on Ki-67 LI cutoff. The whiskers show a 95% confidence interval of the hazard ratios, and the size of the bubbles represents the combined weight of the studies of the respective groups in the meta-analysis.*

Table 5, Covariables included in the respective multivariable cox models.

| **Author** | **Covariables in multivariable Cox models** |
| --- | --- |
| Chen et al. ^3^ | IDH-status, EOR, radiotherapy, microvascular fractal dimension. |
| De Godoy et al. ^4^ | Gender, age, MGMT, EGFRvIII. |
| Fudaba et al. ^5^ | rCBF, ADC ratio, NAA/Cho. |
| Hayashi et al. ^6^ | Temozolomide, high TOPK. |
| Henker et al. ^7^ | Age, KPS, radiotherapy, chemotherapy, edema-tumor ratio. |
| Katsigiannis et al. ^8^ | Age, midline structure involvement, residual tumor volume, MGMT, duration of symptoms, gender. |
| Kim et al. ^9^ | Age, EGFRvIII, preoperative KPS, EOR. |
| Kumar et al. ^2^ | Age, KPS, seizures at presentation, tumor size, EOR, chemoradiation, adjuvant chemotherapy, Stupp protocol, IDH-status, ATRX, tp53. |
| Liu et al. | Postop KPS, extent of resection, adjuvant treatment. |
| Tini et al. ^10^ | Age, KPS, surgery, radiotherapy total dose, radiological treatment response, MGMT-status. |
| Yu et al. | EOR (after stepwise multiple backwards cox regression analysis with the covariables p53, radiotherapy, concurrent temozolomide, adjuvant temozolomide age, sex, tumor origin, tumor location, tumor size, number of lesions, epileptic seizures, intracranial hypertension.) |

Table 6, Median overall survival in months by Ki-67 LI.

| **Author** | **Low Ki-67** | **High Ki-67** | **Difference** |
| --- | --- | --- | --- |
| Chen et al. ^3^ | Not reported |  |  |
| De Godoy et al. ^4^ | 21.1 | 19.8 | 1.3 |
| Fudaba et al. ^5^ | 36.9 | 17.8 | 19.1 |
| Guo et al. ^11^ | 19.3 | 11.6 | 7.7 |
| Hayashi et al. ^6^ | Not reported |  |  |
| Henker et al. ^7^ | 18.2* | 12.4* | 5.8 |
| Katsigiannis et al. ^8^ | Not reported |  |  |
| Kim et al. ^9^ | 21.0 | 20.0 | 1.0 |
| Kumar et al. ^2^ | 14 | 9 | 5 |
| Liu et al. ^12^ | 12.8 | 7.5 | 5.3 |
| Makino et al. | Not reported |  |  |
| Sugita et al. ^13^ | Not reported |  |  |
| Tini et al. ^10^ | 40 | 11 |  |
| Yu et al. | Not reached* | 17.5* |  |

*Estimated from Kaplan-Meier curves using *WebPlotDigitizer* and the *IPDfromKM* package in R.^14,15^

Table 7, Proportion of IDH-wildtype glioblastomas in the respective studies.

| **Author** | **N** | **N IDH-wildtype** | **Proportion IDH-wildtype** |
| --- | --- | --- | --- |
| Chen et al. ^3^ | 94 | 74 | 79% |
| De Godoy et al. ^4^ | 17 | 17 | 100% |
| Fudaba et al. ^5^ | 35 | 30 | 86% |
| Guo et al. ^11^ | 45 | 45 | 100% |
| Hayashi et al. ^6^ | 32 | 31 | 97% |
| Henker et al. ^7^ | 152 | 152 | 100% |
| Katsigiannis et al. ^8^ | 151 | 151 | 100% |
| Kim et al. ^9^ | 20 | 18 | 90% |
| Kumar et al. ^2^ | 160 | 144 | 90% |
| Liu et al. ^12^ | 68 | 65 | 96% |
| Makino et al. | 270 | 270 | 100% |
| Sugita et al. ^13^ | 41 | 41 | 100% |
| Tini et al. ^10^ | 119 | 119 | 100% |
| Yu et al. | 45 | 40 | 100% |
| **Total cohort** | **1249** | **1197** | **96%** |

Table 8, The reported method for assessment of IDH mutation status.

| **Author** | **IDH mutation assessment** |
| --- | --- |
| Chen et al. ^3^ | “Immunohistochemical (IHC) staining was conducted following the standard procedures adapted to antibodies’ protocols. The primary antibodies are against CD34 (Dako, Glostrup, Denmark), Ki67 (Dako), IDH1 R132H (Maixin Bio, Fuzhou, China), and LDHA (Cell Signaling Technology, Beverly, MA). Visualization was achieved by Dako REAL™ EnVision™ Detection System (Dako). Isotype IgGs were used as negative controls.” |
| De Godoy et al. ^4^ | “Tumor specimens were originally cut, mounted, and stained with hematoxylin–eosin by standard methods. The status of isocitrate dehydrogenase (IDH), O6-methylguanine-DNA methyltransferase (MGMT), and EGFRvIII was determined using standard procedures^16^ by a board-certified neuro pathologist (MPN) who was blinded to the neuroimaging results.” |
| Fudaba et al. ^5^ | “For immunostaining, the specimens were sliced from formalin f ixed, paraffin-embedded tissues. We stained the sections with a mouse anti-IDH1 R132H monoclonal antibody (DIA-H09; Dianova GmbH, Hamburg, Germany; 1:20 dilution) (…)” |
| Guo et al. ^11^ | Genomic DNA from formalin-fixed paraffin-embedded (FFPE) tissue was isolated using the DNA FFPE Tissue Kit (Qiagen #56404). A custom glioma next-generation sequencing (NGS) panel was designed to investigate detailed molecular alterations in these patients. The 60-gene NGS panel contains some interested well known genes and recently described genes that were associated with the diagnosis, grading, and treatment responses of gliomas, including IDH1, IDH2 (…) According to the manufacturer’s specifications, The DNA library was constructed by DNA repair (NEBNext® FFPE DNARepairMix,#M6630S), DNA fragmentation and end-repair (TIANSeq Fragment/Repair/Tailing Module, #NG301), and hybridization (KAPA HyperCapture Reagent Kit, #09075828001). (..) After the quality assessment, the library was sequenced with the Illumina platform and the mean read depth was >500X. GRCH38 was used as the reference genome and subsequent analyses |
| Hayashi et al. ^6^ | “The specimens were fixed in buffered formalin (4%) and were embedded in paraffin. Then, 4-μm-thick sections were prepared for the subsequent staining steps. Sections were deparaffinized and heated to retrieve antigenicity. (…) To investigate the relationship between TOPK expression and IDH1 mutation in patients with GBM, the sections were heated to retrieve antigenicity and then anti-IDH1 R132H antibody (1:20; Cosmo Bio, Tokyo, Japan) was applied for 1 h. Then, they were incubated with a biotinylated secondary antibody for 30 min at room temperature, and were stained with DAB and counterstained with hematoxylin.” |
| Henker et al. ^7^ | “Isocitrate dehydrogenase mutation status was determined via Sanger sequencing. If not initially available, analysis was performed retrospectively.” |
| Katsigiannis et al. ^8^ | “IDH-mutation status was detected using immunohistochemistry labeling followed by IDH hotspot sequencing.” |
| Kim et al. ^9^ | “Immunohistochemical reactions were conducted on 4-mm-thick sections of formalin-fixed, paraffin-embedded tissue blocks of 1 representative tumor section. Sections were deparaffinized in xylene and rehydrated in graded alcohols, followed by antigen retrieval in boiling citrate buffer. The endogenous peroxidase activity was blocked using Hydrogen Peroxide Block (Thermo Fisher Scientific, Fremont, CA). Sections were then placed in an automated immunohistochemical stainer (Lab Vision Autostainer LV 1; LabVision/Neomarkers, Fremont, CA) and incubated for 1 hour at room temperature with mouse monoclonal anti-human IDH1R132H antibody (1: 40; clone H09; Dia nova GmbH, Hamburg, Germany) (…)” |
| Kumar et al. ^2^ | “IHC markers were applied over the tumor tissue blocks to determine the mutational status including IDH1-R132H, ATRX loss, TP53 over- expression and Ki-67 index. IHC was done on formalin fixed tissue sections on a Ventana Benchmark LT automated immunostainer (Ven tana Medical Systems, Tucson AZ, USA) according to standard protocols.” |
| Liu et al. ^12^ | “(…) the patient pathology reports were reviewed to obtain molecular testing results, including O6-methylguanine-DNA methyltransferase (MGMT)promoter methylation status, isocitrate dehydrogenase (IDH) status, and Ki67 proliferation index (Ki67 index).” The method for assessment of IDH was not elaborated any further. |
| Makino et al. | “NGS was performed using an amplicon-based glioma-tailored 48-gene (version 2) or 50-gene panel (version 3) (QIA GEN, Hilden, Germany) with 2244 primers for the regions of interest (161,179 bp) and an average exon coverage of 99.95% [12, 13]. The amplicon sequences were aligned to the human reference genome GRCh37 (hg19) in the target region of the sequence. Data were analyzed using the QIA GEN Web Portal service ([https://www.qiagen.com/us/shop/genes-and-pathways/data-analysis-center- overview-page/](https://www.qiagen.com/us/shop/genes-and-pathways/data-analysis-center-%20overview-page/)).” |
| Sugita et al. ^13^ | “Immunohistochemical studies were performed on paraffin sections following heat-induced antigen retrieval, staining with the appropriate antibodies, and signal detection with immunoperoxidase methods (ChemMate ENVISION kit/HRP [DAB], DakoCytomation, Carpinteria, CA, USA) using an autostainer (Dako autostainer universal staining system). Primary antibodies were directed towards (…) IDH-1 R132H (monoclo nal, Dianova, Hamburg, Germany; dilution 1:70), IDH-2 EPR7577 (monoclonal, Abcam, Cambridge, GBR; dilution 1:500) (…)” |
| Tini et al. ^10^ | Not reported. |
| Yu et al. | Not reported. |

Table 9, The reported diagnostic criteria.

| **Author** | **Diagnostic criteria** |
| --- | --- |
| Chen et al. ^3^ | “Pathological diagnoses of all glioma cases were reexamined independently by three neuropathologists (Bian XW, Ping YF, and Xiao HL), according to the WHO Classification of tumors of the Central Nervous System (2016).” |
| De Godoy et al. ^4^ | “(…) all patients (1) had histologically confirmed diagnosis of glioblastoma ^17^ (…)” according to the 2021 WHO classification of CNS tumors. |
| Fudaba et al. ^5^ | “(…) newly diagnosed supratentorial glioblastoma were included in this study. (…) A histologic analysis was performed according to the World Health Organization brain tumor classification revised in 2016 for tissue samples obtained at the time of either surgical resection or image-guided biopsies.” |
| Guo et al. ^11^ | “Hist-GBMs were defined as IDH-wildtype and H3-wildtype high-grade diffuse gliomas with microvascular proliferation and/or intratumoral necrosis. Mol-GBMs were defined as histological WHO grade 2-3 IDH-wildtype and H3-wildtype diffuse gliomas with TERT promoter mutation, EGFR amplification, or +7/-10 chromosome variations.” |
| Hayashi et al. ^6^ | Histopathological diagnosis of glioblastoma multiforme. Not otherwise specified. Patient inclusion was conducted from 2006-2015, before the 2016 WHO classification. The study was published in 2017. |
| Henker et al. ^7^ | “(…) newly diagnosed and histologically proven IDH wild-type glioblastoma were included (…) Definition of IDH wild-type glioblastoma was based on isocitrate dehydrogenase 1 R132 mutation status.” |
| Katsigiannis et al. ^8^ | “(…) primary GBM (…) Neuropathological diagnosis was performed in accordance with the 2016 WHO classification and patients with a mutation encoding for the isocitrate dehydrogenase (IDH) were not included, since they most probably represented secondary GBM with a completely different prognosis” |
| Kim et al. ^9^ | Histopathologically confirmed glioblastoma multiforme. Diagnostic criteria were not otherwise specified. Patients were treated between 1996 and 2010. The study was published in 2012. |
| Kumar et al. ^2^ | “The diagnosis of glioblastoma was made pathologically, and grading was done based on WHO classification.” Patients were included between 2012 and 2018. The study was published in 2021. |
| Liu et al. ^12^ | “(…) elderly glioblastoma patients (aged ≥ 60 years old at the time of operation) treated between January 2015 and December 2019. Patients were excluded from the study if they had recurrent lesions or received preoperative chemoradiotherapy.” Diagnostic criteria or WHO classification not otherwise specified. |
| Makino et al. | “From a multi-institutional glioma cohort, we collected 270 samples from adult patients newly diagnosed with GBM, IDH-wildtype, WHO grade 4 between 2011 and 2023. All cases were diagnosed on the basis of the 2021 WHO classification of tumors of the CNS. Patients with diffuse midline glioma with H3-K27M mutation, loss of H3K27me3, or overexpression of EZHIP were excluded. The tumors were fixed with phosphate-buffered 10% formalin for 24 h, embedded in paraffin, and sectioned for hematoxylin and eosin staining. Board-certified pathologists histologically evaluated all tissues to ensure an estimated tumor cell content of 30% or more.” |
| Sugita et al. ^13^ | “Tissue samples were fixed in 20% buffered formalin and embedded in paraffin. In 30 of the 41 cases, tissue micro-arrays were constructed and processed using conventional histological and immunohistochemical methods. In the remaining nine samples, in which tissue array construction was difficult, conventional sections (5-μm thickness) were stained with hematoxylin and eosin (HE) for histological evaluation, and the remaining serial unstained sections were used for immunohistochemistry. Due to the heterogeneous nature of GB, in all cases, whole sections of the excised tumor were taken to prepare tissue specimens. Each tissue specimen was microscopically examined, and the sections with the richest concentration of tumor components were used. All specimens were histologically diagnosed according to the [2021] WHO criteria for tumors of the CNS.^17^” |
| Tini et al. ^10^ | “Between February 2016 and July 2021, 183 consecutive patients with GBM were treated at University Hospital of Siena, Italy.” The diagnostic criteria were not otherwise specified. |
| Yu et al. | “The GSM [gliosarcoma] was diagnosed according to the biphasic growth pat tern of hematoxylin-eosin (HE) staining as well as GFAP staining demonstrating GFAP-positive glioma components and GFAP-negative sarcoma components containing tumor spindle cells (Figures 1(c)–1(f)). The pathological specimens before the release of 2016 World Health Organization Classification of Tumors of the Central Nervous System were reviewed and confirmed by the pathologist. Radiologic and pathologic findings were read and examined by specialists who did not know patient information following the principle of blindness.” |

References

1. Tini P, Yavoroska M, Mazzei MA, et al. Low expression of Ki-67/MIB-1 labeling index in IDH wild type glioblastoma predicts prolonged survival independently by MGMT methylation status. Article. *J Neurooncol*. 2023;163(2):339-344. doi:10.1007/s11060-023-04342-2

2. Kumar N, Elangovan A, Madan R, et al. Impact of Immunohistochemical profiling of Glioblastoma multiforme on clinical outcomes: Real-world scenario in resource limited setting. *Clin Neurol Neurosurg*. Aug 2021;207:106726. doi:10.1016/j.clineuro.2021.106726

3. Chen C, He ZC, Shi Y, et al. Microvascular fractal dimension predicts prognosis and response to chemotherapy in glioblastoma: an automatic image analysis study. *Lab Invest*. Jul 2018;98(7):924-934. doi:10.1038/s41374-018-0055-2

4. de Godoy LL, Chawla S, Brem S, et al. Assessment of treatment response to dendritic cell vaccine in patients with glioblastoma using a multiparametric MRI-based prediction model. *J Neurooncol*. May 2023;163(1):173-183. doi:10.1007/s11060-023-04324-4

5. Fudaba H, Momii Y, Matsuta H, et al. Perfusion Parameter Obtained on 3-Tesla Magnetic Resonance Imaging and the Ki-67 Labeling Index Predict the Overall Survival of Glioblastoma. *World Neurosurg*. May 2021;149:e469-e480. doi:10.1016/j.wneu.2021.02.002

6. Hayashi T, Hayakawa Y, Koh M, et al. Impact of a novel biomarker, T-LAK cell-originating protein kinase (TOPK) expression on outcome in malignant glioma. *Neuropathology*. Apr 2018;38(2):144-153. doi:10.1111/neup.12446

7. Henker C, Kriesen T, Schneider B, et al. Correlation of Ki-67 Index with Volumetric Segmentation and its Value as a Prognostic Marker in Glioblastoma. *World Neurosurg*. May 2019;125:e1093-e1103. doi:10.1016/j.wneu.2019.02.006

8. Katsigiannis S, Krischek B, Barleanu S, et al. Impact of time to initiation of radiotherapy on survival after resection of newly diagnosed glioblastoma. *Radiation Oncology*. Apr 2019;1473. doi:10.1186/s13014-019-1272-6

9. Kim JH, Bae Kim Y, Han JH, et al. Pathologic diagnosis of recurrent glioblastoma: Morphologic, immunohistochemical, and molecular analysis of 20 paired cases. Article. *American Journal of Surgical Pathology*. 2012;36(4):620-628. doi:10.1097/PAS.0b013e318246040c

10. Tini P, Yavoroska M, Mazzei MA, et al. Low expression of Ki-67/MIB-1 labeling index in IDH wild type glioblastoma predicts prolonged survival independently by MGMT methylation status. *J Neurooncol*. Jun 2023;163(2):339-344. doi:10.1007/s11060-023-04342-2

11. Guo X, Gu L, Li Y, et al. Histological and molecular glioblastoma, IDH-wildtype: a real-world landscape using the 2021 WHO classification of central nervous system tumors. Article. *Front Oncol*. 2023;13doi:10.3389/fonc.2023.1200815

12. Liu J, Li C, Wang Y, et al. Prognostic and Predictive Factors in Elderly Patients With Glioblastoma: A Single-Center Retrospective Study. *Front Aging Neurosci*. 2021;13:777962. doi:10.3389/fnagi.2021.777962

13. Sugita Y, Furuta T, Takahashi K, et al. Elevated expression of N-myc downstream regulated gene 1 protein in glioblastomas reflects tumor angiogenesis and poor patient prognosis. *Neuropathology*. Dec 2024;44(6):422-431. doi:10.1111/neup.12999

14. *WebPlotDigitizer*. Version 5.2. <https://automeris.io>

15. *IPDfromKM: Map Digitized Survival Curves Back to Individual Patient Data*. Version 0.1.10. 2020. <https://CRAN.R-project.org/package=IPDfromKM>

16. Nasrallah MP, Binder ZA, Oldridge DA, et al. Molecular Neuropathology in Practice: Clinical Profiling and Integrative Analysis of Molecular Alterations in Glioblastoma. *Academic Pathology*. 2019;6:237428951984835. doi:10.1177/2374289519848353

17. Louis DN, Perry A, Wesseling P, et al. The 2021 WHO Classification of Tumors of the Central Nervous System: a summary. *Neuro Oncol*. Aug 2 2021;23(8):1231-1251. doi:10.1093/neuonc/noab106
