# Supplementary figures and images for: Ki-67 and overall survival in patients with glioblastoma: A systematic prognostic review and meta-analysis
Source: Neurooncol Adv. 2026 Apr 27;8(1):vdag111. doi: 10.1093/noajnl/vdag111 (PMC13213614; doi:10.1093/noajnl/vdag111)

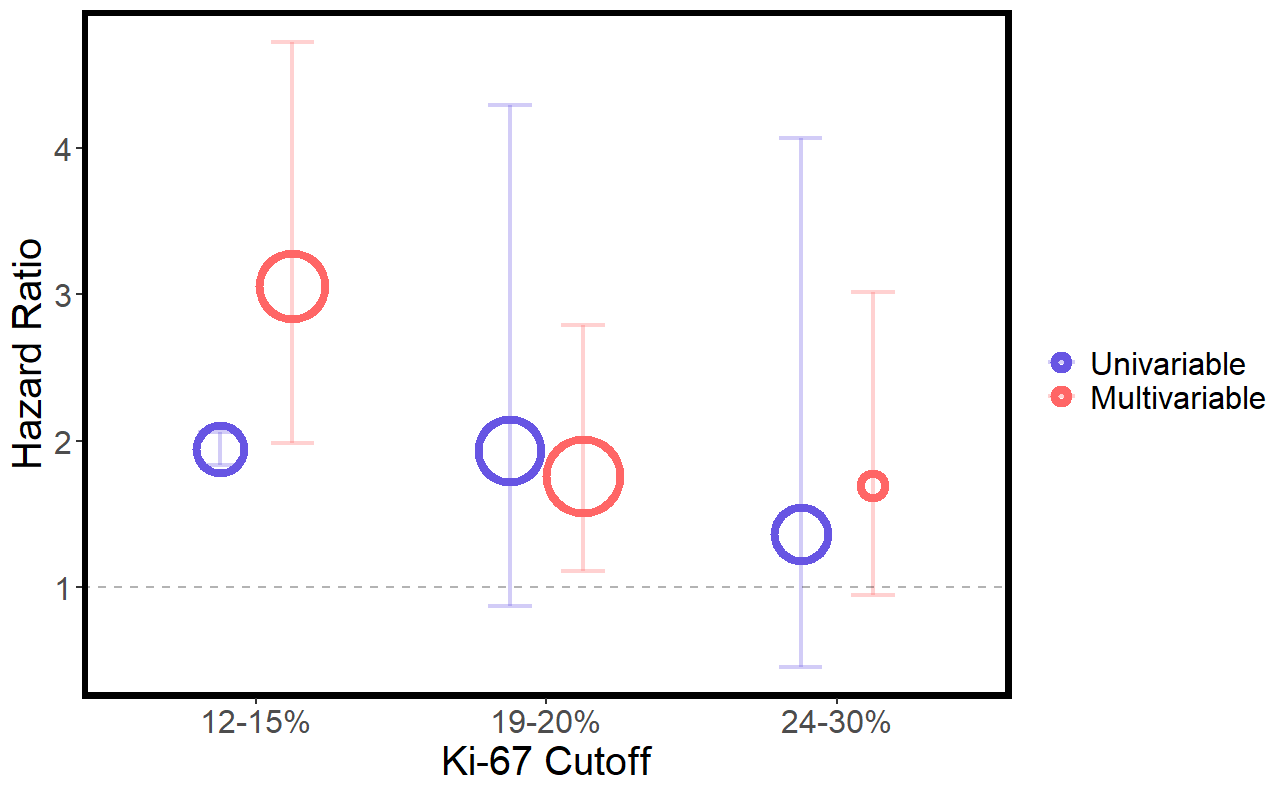

Supplement: vdag111_Supplementary_Data [file vdag111_supplementary_data.zip › Figure S8.tiff]

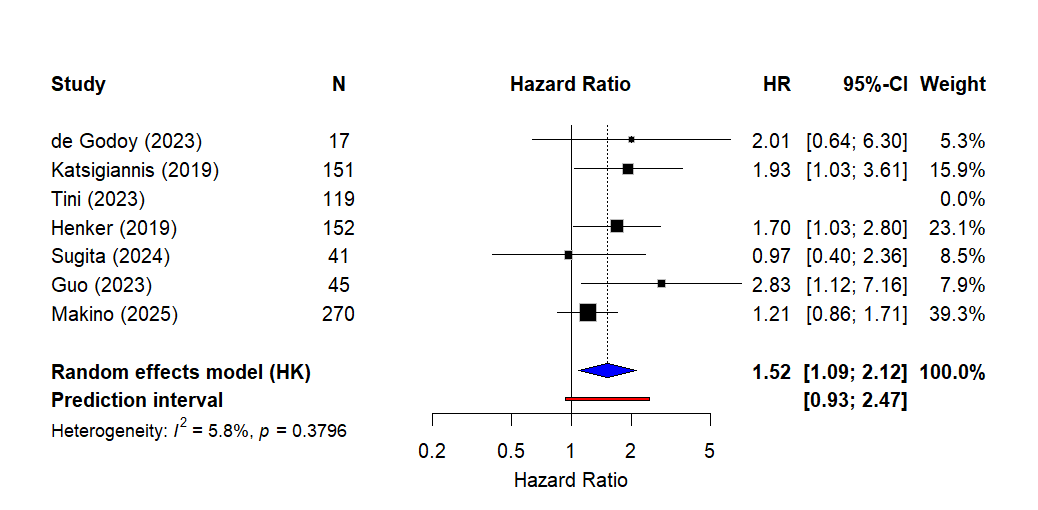

Supplement: vdag111_Supplementary_Data [file vdag111_supplementary_data.zip › Figure S1.tiff]

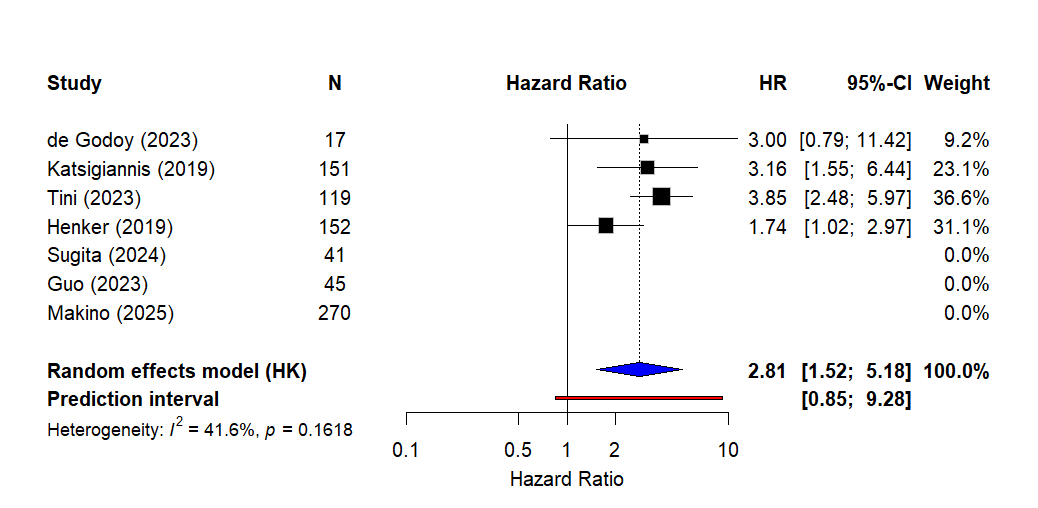

Supplement: vdag111_Supplementary_Data [file vdag111_supplementary_data.zip › Figure S2.tiff]

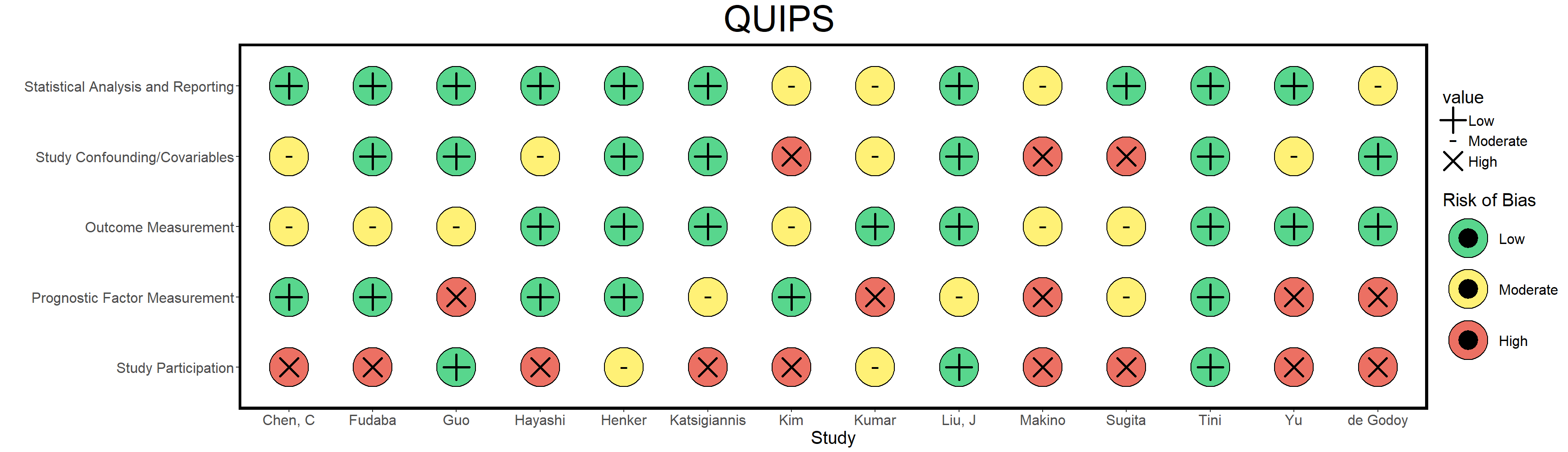

Supplement: vdag111_Supplementary_Data [file vdag111_supplementary_data.zip › Figure S3.tiff]

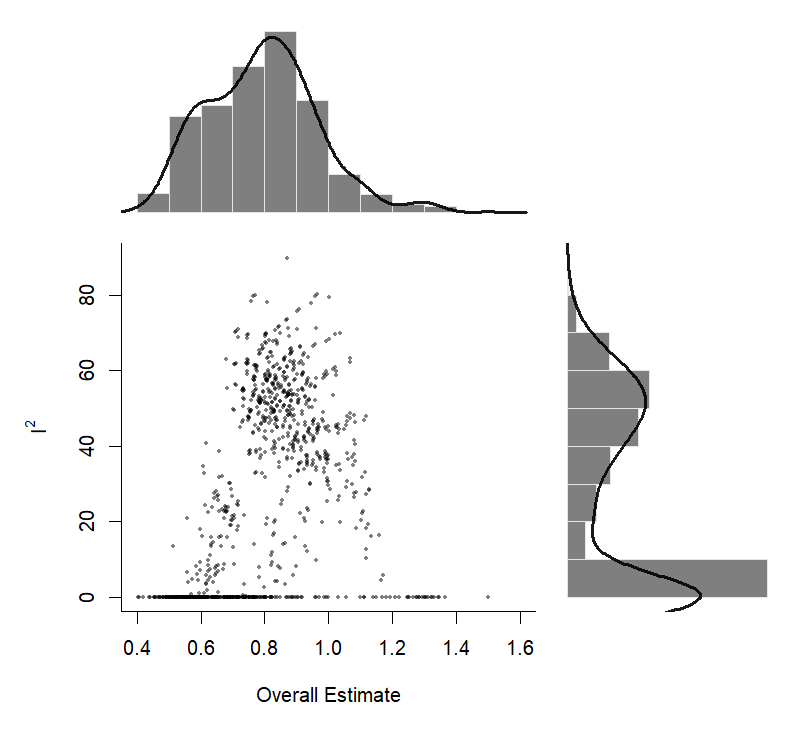

Supplement: vdag111_Supplementary_Data [file vdag111_supplementary_data.zip › Figure S4.tiff]

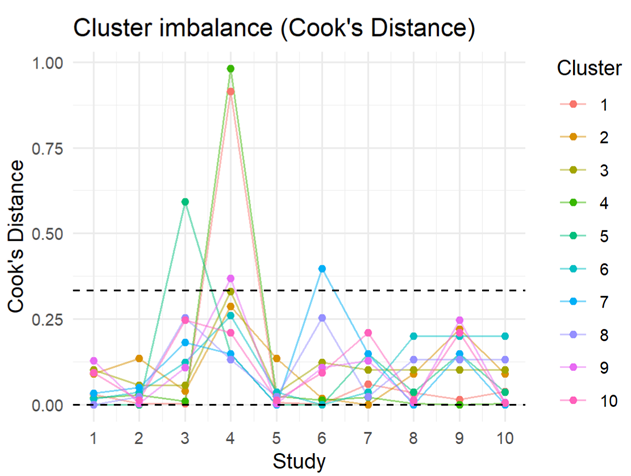

Supplement: vdag111_Supplementary_Data [file vdag111_supplementary_data.zip › Figure S5.tif]

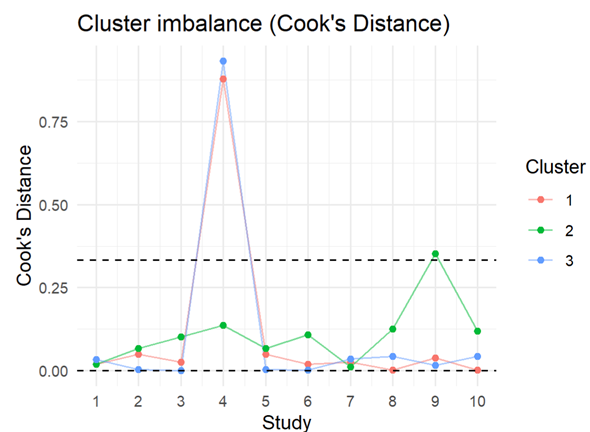

Supplement: vdag111_Supplementary_Data [file vdag111_supplementary_data.zip › Figure S6.tif]

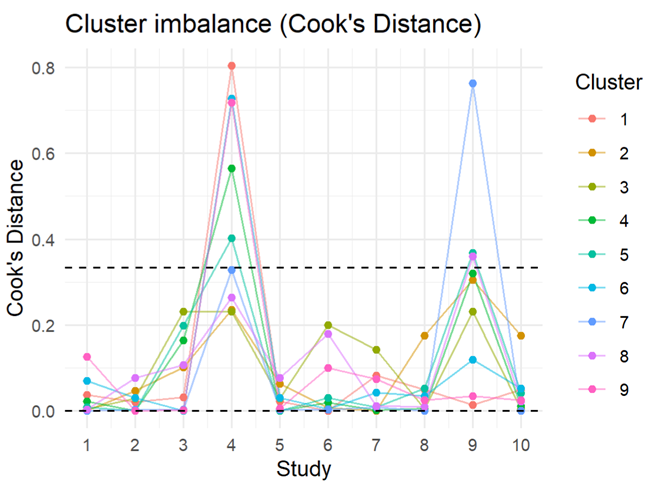

Supplement: vdag111_Supplementary_Data [file vdag111_supplementary_data.zip › Figure S7.tif]
